# The Association between Internet Use and Physical Exercise among Middle-Aged and Older Adults—Evidence from China

**DOI:** 10.3390/ijerph192416401

**Published:** 2022-12-07

**Authors:** Bin Guo, Xiaodong Zhang, Rui Zhang, Gong Chen

**Affiliations:** 1 Institute of Population Research, Peking University, Beijing 100871, China; 2 Department of Physical Education, Peking University, Beijing 100871, China

**Keywords:** Internet use, physical exercise, population aging, middle-aged and older adults, China

## Abstract

Background: In an aging and digital society, Internet use is significantly associated with residents’ physical exercise. This study aimed to explore the association between Internet use and physical exercise among Chinese middle-aged and older adults in two respects: Internet use and the purpose of Internet use. Methods: The data used in this study were obtained from the 2018 China Health and Retirement Longitudinal Study (CHARLS) conducted by Peking University. The logit model and the ordered probit model were used to analyze the association between Internet use and physical exercise, and the substitution variable method was used to examine the robustness of the results. Results: (1) Internet use and the frequency of Internet use significantly increased the probability and frequency of middle-aged and older adults’ participation in physical exercise (*p* < 0.001). (2) “Watching news”, “chatting” and “watching videos” via the Internet were positively associated with physical exercise, while “playing games” had no impact. (3) Internet use had a greater impact on physical exercise participation among middle-aged adults and those living in urban areas than among older adults and those living in rural areas. Conclusions: This study suggests that Internet use among middle-aged and older adults is positively associated with their participation in physical exercise; the government should try to increase the popularity of Internet use to encourage their participation in physical exercise.

## 1. Introduction

The increasing aging trend is gradually becoming an important issue for most countries in the 21st century. According to data released by the United Nations, the aging trend will continue to deepen globally, with the proportion of the global population aged 65 and above due to rise to 16% in 2050 [1]. Unfortunately, the Chinese government faces an even greater challenge. The data released by the National Bureau of Statistics show that the number of people aged 60 and older has reached 264.02 million, accounting for 18.7% of the total population [2]. In order to better cope with the possible health problems caused by population aging, the Chinese government issued the “National Medium and Long-term Plan for Actively Coping with Population Aging”, which elevates coping with population aging to a national strategy. Encouraging middle-aged and older groups to participate in physical exercise is an important approach to actively cope with population aging. Numerous studies have shown that physical exercise can significantly improve the physical and mental health and social participation of older people [3,4,5,6,7], and thus, improve their overall health and wellbeing. Some European countries proposed the slogan “Sport for All” to actively promote physical fitness in middle-aged and older adults to improve their health status [8,9], and the Chinese government also included the increase in physical exercise participation rate in the “National Fitness Program” and “Outline for Building a Leading Sports Nation” [10].

Regarding the factors affecting physical exercise, the literature mainly explores macro and micro aspects. At the macro level, the level of regional economic development, the accessibility of physical exercise venues and the availability of scientific fitness guidance have a significant impact on individuals’ physical exercise. For example, since urban areas have better economies and more convenient sports facilities than rural areas, older adults living in cities were more likely to participate in physical exercise than those living in rural areas [11,12]. Moreover, several studies showed that a lack of venue facilities is another vital factor affecting older adults’ participation in physical exercise [13,14]. At the micro level, an individual’s education level, health status, lifestyle, income level and awareness of healthcare can also have an important impact on their adoption of physical exercise [15,16]. The higher a resident’s education level [11,17], awareness of healthcare and household income [18], the more frequently they participate in physical exercise.

With the advance of science and technology, we are now living in a digital society. One of the basic features of this society that has emerged after the dominance of agricultural and industrial societies is the popularity and widespread use of the Internet. According to data released by China Internet Network Information Center, the number of Internet users aged 40 and older in China has reached 467 million, and the Internet penetration rate among people aged 60 and above has reached 43.2%, which is expected to continue to rise. By using the Internet, middle-aged and older adults can encounter a large amount of information, instant interactions and a variety of convenient services without having to leave home. The Internet is a major source of health information [19]. Many studies have shown that Internet use has a vital impact on the health status (physical and mental health), social participation and social integration of older adults. For example, Cohall et al. (2011) found that Internet use can lead individuals to gain more knowledge regarding a variety of health-related topics, which in turn can improve their physical health [20,21]. White et al. (2002) concluded that Internet use can significantly reduce loneliness and increase life satisfaction among older adults [22,23], more importantly, Internet use can facilitate older adults’ communication with their friends and participation in online leisure activities, thereby increasing their level of social participation [24,25,26]. Roberta et al. (2021) found that fostering eHealth literacy in older adults offered the potential to improve the wellbeing of older people in today’s rapidly developing society [27].

In this context, the association of Internet use and residents’ physical exercise has gradually become a major research topic [28,29,30,31,32]. Some scholars argue that Internet use encourages physical exercise among older adults by increasing individuals’ access to information [31], facilitating community communication and enhancing the knowledge of exercise and sports. For example, Wang et al. (2021) found that Internet use had a significant impact on increasing physical exercise by using the 2017 China General Social Survey data [33]. Liu et al. (2021) used the same data to establish that the targeted Internet pushing of health and exercise-related information could encourage physical exercise [34]. However, other scholars reached different conclusions; they believed that Internet use would reduce leisure time and time that would otherwise be spent on exercise and sleep, thereby having a suppressive impact on the frequency of physical exercise participation among the elderly. For example, Li et al. (2022) used the 2018 CFPS data and found Internet use significantly reduced the duration and frequency of physical exercise among rural residents aged 40 and above [35].

However, previous studies mainly focused on the relationship between Internet use and physical exercise. There are still some limitations. First, the studies explored the association between Internet use and physical exercise of residents, while ignoring the impact of the specific purpose of Internet use on individual physical exercise. More importantly, little research discusses the association mechanisms of Internet use with physical exercise, and more empirical evidence in this area is needed. In response to these questions, this paper uses the 2018 CHARLS data to provide insights into the relationship between Internet use and physical exercise among middle-aged and older adults in the context of aging and digitalization, and further explore the possible impact mechanisms. The possible research contributions of this paper are as follows. First, in this study, we refine the measurement indicators of Internet use. We not only analyze the effects of Internet use and the frequency of Internet use on middle-aged and older adults’ physical exercise, but also further analyze the association between Internet-use purpose and physical exercise. This multidimensional analysis can provide a reference for future government policies related to Internet use and participation in physical exercise. Second, the literature hardly explores the association mechanism of Internet use with physical exercise among middle-aged and older adults, and this paper attempts to explore the association mechanism in terms of two effects (time-crowding-out effect and willingness-to-crowd-in effect). Finally, this paper also analyzes the possible age and urban–rural differences in the association between Internet use and physical exercise.

## 2. Methods

### 2.1. Data

The data used in this paper are from the China Health and Retirement Longitudinal Study (CHARLS), published by the China Social Science Research Center of Peking University in 2018. CHARLS is a set of high-quality micro-data representing Chinese middle-aged and older households and individuals aged 45 years and older. The database covers 150 county-level units, 450 village-level units and 19,000 people in approximately 10,000 households nationwide. The CHARLS database mainly contains modules such as “Demographic Backgrounds”, “Family”, “Health Status and Functioning”, “Health Care and Insurance”, “Work and Retirement”, “Pension”, “Income, Expenditures and Assets” and “House Property and Housing Characteristics”. It is one of the high-quality and large micro-tracking databases that are publicly available in China. According to our research needs, we cleaned the data as follows. Firstly, we matched and merged all modules and removed the duplicate values. Secondly, considering that disability and physical-function impairment may limit middle-aged and older adults’ participation in physical exercise, samples with disabilities were removed. Finally, missing data of important variables were removed, and extreme values of income variables and data with “do not know” and “refused to answer” for each indicator were excluded. The final sample size for analysis was 11,858.

### 2.2. Variable Measurement

Dependent variable. Referring to the definition and measurement indicators of physical exercise by the Chinese government and in combination with the design of the 2018 CHARLS questionnaire [36,37], we selected two indicators, “whether to participate in physical exercise” and “frequency of participating in physical exercise”, to measure the physical exercise of the middle-aged and older adults. First, we chose the question, “do you usually take part in exercise for at least 30 min every week?”, to measure the physical exercise of the interviewees. When the interviewees had exercised at least 30 min weekly, this was categorized as “1”, otherwise it was categorized as “0”. Second, we chose the question, “how many days a week do you take part in exercise for at least 30 min?” to measure the frequency of physical exercise; the interviewees needed to choose from 1–7 days. When a respondent selected “1–2 days”, this was defined as “1 = not regularly”; when a respondent selected “3–4 days”, this was defined as “2 = regularly”; when the respondent selected “5 days and above”, this was defined as “3 = almost daily”; and when the respondent did not participate in physical exercise, this was defined as “0 = never”.

Independent variable. The core independent variable of this study was the Internet-use status of the middle-aged and older adults. According to the literature and the design of the 2018 CHARLS questionnaire, we chose three indicators, “whether to use the Internet”, “the frequency of Internet use” and “the purpose of Internet use”, to measure the Internet-use status of the respondents [21]. First, we chose the question, “have you used the Internet in the last month?”, to measure the Internet use of the respondents. When the respondents answered “yes”, the value was 1; otherwise, the value was 0. Second, we chose the question, “how often in the last month did you use the Internet? Almost daily, almost every week, not regularly or never?”, to measure the individual’s Internet use frequency. When the respondent selected “never”, “not regularly”, “almost every week” or “almost daily”, they were assigned 0, 1, 2 and 3, respectively. Third, we chose the question, “what do you usually do on the Internet?” to measure the respondents’ Internet-use purpose. The following options were provided in the questionnaire: “chat”, “watch news”, “watch videos” and “play games”. We generated the dummy variable of Internet-use purpose. The specific definitions are presented in Table 1.

Covariates. To reduce the possible bias in the statistical model due to omitted variables, some variables were controlled for in the empirical analysis: individual characteristic variables including gender, age, marital status, household registration nature and nationality; human-capital status, including individual education level, self-rated health, mental health level and number of chronic diseases, and social and economic indicators, including family income level, pension insurance, medical insurance participation and work status. Meanwhile, considering that differences and imbalances in Internet penetration among different provinces and cities in China may have exerted an impact on residents’ physical exercise, in the empirical analysis, we controlled for the provinces where the middle-aged and older adults were located. Specific definitions and descriptive statistics are presented in Table 1.

### 2.3. Analysis Method

STATA version 16.0 was used to analyze the association between Internet use and physical exercise among middle-aged and older adults. Logit model was used to analyze the association between Internet use and physical exercise of middle aged and older people, and ordered probit model was used to evaluate the association between Internet use and frequency of physical exercise. Moreover, the substitute variable method was adopted to check the robustness of the regression results.

## 3. Results

### 3.1. The Association between Internet Use and Physical Exercise of Middle-Aged and Older Adults

Column (1) in Table 2 reports the regression results of the association of Internet use and the physical exercise. The results show that Internet use can significantly increase the probability of physical exercise of middle-aged and older adults. The probability of participating in physical exercise among middle-aged and older adults who use the Internet was 5.1% higher than that of those who did not use the Internet (*p* < 0.001). Column (2) in Table 2 reports the association between Internet use and the frequency of physical exercise. The regression results show that Internet use can increase the frequency of physical exercise. The frequency of physical exercise among middle-aged and older adults using the Internet was higher than among those who did not use Internet (β = 0.117, *p* < 0.001). Columns (3)–(4) in Table 2 report the results of the influence of Internet-use frequency on physical exercise among the middle-aged and older adults. The results show that the higher the frequency of Internet use among the middle-aged and older adults, the higher the probability (marginal effect = 0.017, *p* < 0.001) and frequency of them participating in physical exercise (β = 0.061, *p* < 0.001).

From the perspective of the controlled variables, among the individual characteristic variables, Hukou and smoking were negatively associated with physical exercise (*p* < 0.01). Among the human-capital variables, the higher the education level and the more chronic diseases the participants suffered from, the higher the probability and frequency of their participation in physical exercise (*p* < 0.001). However, the self-rated health and mental health were negatively related to their participation in physical exercise. The worse the self-rated health (*p* < 0.05) and mental health (*p* < 0.001), the lower the probability and frequency of participating in physical exercise. Finally, the social and economic level of the middle-aged and older adults were also positively associated with their participation in physical exercise. The higher their household income, the more likely the adults were to participate in physical exercise (*p* < 0.05). Compared with the agricultural self-employed middle-aged and older adults, the retired and unemployed/out-of-work groups had a higher probability of participating in physical exercise (*p* < 0.001).

### 3.2. The Association between Internet-Use Purpose and Physical Exercise among Middle-Aged and Older Adults

In this study, the purpose of Internet use was divided into “chatting”, “watching news”, “watching videos” and “playing games”. Table 3 reports the association between Internet-use purpose and physical exercise among the middle-aged and older adults. Columns (1)–(4) in Table 3 show that using the Internet for “chatting”, “watching news” and “watching videos” can increase the probability of participating in physical exercise by 4.8%, 5.7% and 4.3%, respectively (*p* < 0.001), but using the Internet to play games has no association on their physical exercise. Columns (5)–(8) in Table 3 report the association between Internet-use purpose and the frequency of physical exercise. The results show that middle-aged and older adults who use the Internet for “chatting”, “watching news” and “watching videos” participated in physical exercise more frequently (*p* < 0.001). Similarly, using the Internet to play games had no impact on the frequency of their participation in physical exercise. On the whole, using the Internet to watch news had the greatest impact on physical exercise among the middle-aged and older adults, while using the Internet to play games had no impact on their participation in physical exercise.

### 3.3. Robustness Test

We used the substitute variable method to test the robustness of the basic results. According to the design of the 2018 CHARLS questionnaire, we adopted the questions “does your residence have broadband Internet connection?” and “do you use WeChat?” as a substitute variable for Internet use. Table 4 reports the regression results of the influence of broadband Internet connection and WeChat use on physical exercise among the middle-aged and older adults. The results in columns (1)–(2) show that compared with those who could not access broadband Internet in their residence, the middle-aged and older adults who could access broadband Internet in their residence had a higher probability and frequency of participating in physical exercise (*p* < 0.001). Columns (3)–(4) in Table 4 report the impact of WeChat use on physical exercise among the middle-aged and older adults. The results show that the middle-aged and older adults who use WeChat had a higher probability and frequency of physical exercise than those who did not use WeChat (*p* < 001). The results in Table 4 are consistent with those in Table 2.

### 3.4. Intergenerational Differences and Urban–Rural Differences Regarding the Association between Internet Use and Physical Exercise

Considering that there was a large difference in the Internet penetration rate between different age groups, columns (1)–(4) in Table 5 divide the age of the middle-aged and the elderly into two groups, “45–59 years old” and “60 years older and above”, respectively, and report the differences in the association between Internet use with physical exercise. The results show that Internet use improves the probability and frequency of physical exercise for the middle-aged and elderly groups, but it had a greater impact on the middle-aged groups. Columns (5)–(8) report the urban–rural differences in the association of Internet use with physical exercise. The results show that Internet use significantly improves the probability and frequency of rural and urban residents participating in physical exercise, but it had a greater impact on the urban middle-aged and elderly groups.

## 4. Discussion

Most previous studies explored the impact of Internet use and physical exercise on the physical and mental health and social participation of older adults [38,39,40], while fewer studies systematically explored the associations between Internet use and physical exercise participation. In this paper, we empirically analyzed the relationship between Internet use and physical exercise among the middle-aged and older adults in the context of an aging population and the digital society based on the 2018 CHARLS data. We found: (1) Internet use was positively associated with participation in physical exercise among middle-aged and older adults; the probability and frequency of participation in physical exercise were 5.1% (*p* < 0.001) and 0.177 (*p* < 0.001) higher than those for adults who did not use the Internet, respectively. Moreover, the higher the frequency of Internet use, the higher the probability and frequency of participation in physical exercise. (2) Different Internet-use purposes had different associations on physical exercise; “chatting”, “watching news” and “watching videos” via the Internet were positively associated with physical exercise in middle-aged and older adults, among which “watching news” had the greatest impact on physical exercise, while “playing games” had no impact. (3) Internet use had a greater association on middle-aged adults living in urban areas than on older adults living in rural areas.

First, we found that Internet use and frequency of Internet use were positively associated with middle-aged and older adults’ participation in physical exercise. There are two main findings in previous studies regarding the association between Internet use and physical exercise. The first is that Internet use can significantly increase the probability of residents participating in physical exercise. For instance, Wang et al. (2021) used the 2017 CGSS data and found that older adults who regularly used the Internet were significantly more physically active and performed more moderate-to-vigorous physical exercise per week [33]. Kearns et al. (2019) drew similar conclusions from a survey of the UK community [41]. Other studies reached the opposite conclusion, suggesting that Internet use had a suppressive impact on the frequency of physical exercise participation among older adults. For example, Li et al. (2022) found that Internet use significantly reduced the duration and frequency of physical exercise among rural residents [35]. Sahin (2018) concluded that Internet use is associated with low physical exercise [42].

The findings of our study supported the first view. Internet use and participation in physical exercise are both important forms of active aging [43,44], and we hold that there are two simultaneous and mutually exclusive effects between them: one is the time-crowding-out effect of physical exercise in the digital age since both Internet use and physical exercise occur during leisure time, there is time competition between these two behaviors when leisure time is relatively fixed, creating a disincentivizing effect of Internet use on older adults’ physical exercise. The other is the crowding-in effect of physical exercise willingness due to information empowerment. According to the theory of network empowerment, actors use network participation to enhance their ability and confidence in controlling their lives [45], for example, the middle-aged and older adults could gain more knowledge and guidance on physical exercise through Internet use, which would increase their willingness to participate in physical exercise, thus increasing their participation in and frequency of physical exercise. When the crowding-in impact of physical exercise willingness due to information empowerment is greater than the time-crowding-out impact of physical exercise in the digital age, Internet use has a positive association with the physical exercise of middle-aged and older adults.

Second, we found that different purposes of Internet use had different effects on physical exercise among middle-aged and older adults. Our study found that “watching news”, “chatting” and “watching videos” via the Internet had positive associations on physical-exercise participation, increasing the probability of physical-exercise participation by 5.7%, 4.8% and 4.3%, respectively (*p* < 0.001), but “playing games” had no association on physical exercise. We believe that this result was due to the fact that “chatting”, “watching news” and “watching videos” via the Internet can increase access to sports information and sports-health knowledge, thus increasing users’ willingness to participate in physical exercise, while these Internet activities occupy little leisure time.

Finally, we also found a greater association of Internet use on physical exercise among middle-aged adults and those living in urban areas relative to older adults (60+ years) and those living in rural areas. We believe that middle-aged adults are more proficient at using the Internet than older adults and are able to obtain more information and knowledge about physical exercise via the Internet, thus increasing the frequency of their physical exercise. Compared to those who live in urban areas, middle-aged and older adults in rural areas do not have high Internet-penetration rates, their access to sports facilities is not strong and the importance they attribute to physical exercise is relatively low. Therefore, the association of Internet use on middle-aged and older adults’ physical exercise in rural areas is weaker.

Based on the above analysis, this study proposes the following policy recommendations to strengthen the role of the Internet in encouraging the participation of middle-aged and older adults in physical exercise. First, we should transmit more scientific fitness knowledge and cultivate the awareness of active fitness among middle-aged and older adults via the Internet. Second, middle-aged and older adults should avoid falling into the “internet-addiction trap”, which occupies time that might otherwise be used for physical exercise; this can be achieved by, for example, setting up a long-time-use-reminder function in the Internet applications to prompt proper rest and exercise. Finally, it is necessary to increase training on Internet use and the development of physical exercise methods suitable for older adults with agricultural household registration in rural areas. In particular, when formulating relevant policies, we should focus on considering the placement of Internet and sports resources in rural areas to further encourage the balance of Internet services and public sports facilities in urban and rural areas.

There are some limitations to this study. First, the cross-sectional data of the 2018 CHARLS was used to explore the association between Internet use and physical exercise participation of middle-aged and older adults, so this study essentially was a correlational study. We did not deal with the endogenous problem caused by reverse causality, so we failed to identify the causal relationship between Internet use and participation in physical exercise. Second, due to data limitations, the association mechanisms we mentioned were mainly explored at the theoretical level and lacked empirical tests, and we will continue to conduct relevant research once the data are available. Third, the current study only focuses on Internet use, the purpose of Internet use and the probability and frequency of physical exercise; it lacks indicators of the length (intensity) of Internet use and physical-exercise intensity. We will therefore conduct more in-depth studies in future.

## 5. Conclusions

In conclusion, our study shows that Internet use among middle-aged and older adults was positively associated with their participation in physical exercise. Watching news, chatting and watching videos via the Internet were positively related to physical exercise in the middle-aged and older adults, which may be the result of the interaction between time-crowding-out and willingness-to-crowd-in. Internet use had a greater association on the middle-aged adults who were 45–59 years old and the middle-aged adults living in urban areas than on the older adults (60+ years old) and the middle-aged adults living in rural areas.

## Figures and Tables

**Table 1:** Definition of variables and descriptive statistics.

	Definition of Variables	Frequency/Mean	Percent/SE
Dependent variable	Exercise:		
	No = 0	8731	73.63
	Yes = 1	3127	26.37
	Frequency of exercise:		
	Never = 0	8731	73.63
	Not regularly = 1	198	1.67
	Regularly = 2	207	1.75
	Almost daily = 3	2722	22.95
Independent variable	Internet use:		
	No = 0	9815	82.77
	Yes = 1	2043	17.23
	Frequency of Internet use:		
	Never = 0	9815	82.77
	Not regularly = 1	178	1.50
	Almost every week = 2	158	1.33
	Almost daily = 3	1707	14.40
	Purpose of Internet use:		
	Chat: No = 0	10,749	88.78
	Yes = 1	1359	12.22
	Watch news: No = 0	10,408	85.96
	Yes = 1	1700	14.04
	Watch videos: No = 0	1367	88.71
	Yes = 1	10,741	11.29
	Play games: No = 0	11,631	96.06
	Yes = 1	477	3.94
Control variables	Age:	59.85	9.318
	Gender:		
	Female = 0	6205	52.33
	Male = 1	5653	47.67
	Marital status:		
	No spouse = 0	1877	13.44
	Married with a spouse = 1	12,084	86.56
	Hukou:		
	Urban area = 0	1288	10.86
	Rural area = 1	10,570	89.14
	Ethnicity:		
	Other = 0	839	7.08
	Han Chinese = 1	11,019	92.92
	Smoking:		
	No = 0	7007	59.09
	Yes = 1	4851	40.91
	Drinking:		
	No = 0	7500	63.25
	Yes = 1	4358	36.75
	Education level:		
	Illiterate = 1	4194	35.37
	Elementary = 2	2821	23.79
	Middle school = 3	3026	25.52
	High school and above = 4	1817	15.32
	Self-rated health:		
	Very good = 1	1732	14.61
	Good = 2	1742	14.69
	Fair = 3	6165	51.99
	Poor = 4	1809	15.26
	Very poor = 5	410	3.46
	Mental health: the score of depression	17.64	6.026
	Number of chronic diseases:	1.55	1.150
	Health insurance:		
	No = 0	378	3.19
	Yes = 1	11,480	96.81
	Pension:		
	No = 0	1554	13.11
	Yes = 1	10,304	86.89
	Logarithm of household income:	9.988	1.818
	Occupation:		
	Self-employed agricultural work = 1	3605	30.40
	Employed = 2	2790	23.53
	Retirement = 3	930	7.84
	Non-farm self-employed = 4	1589	13.40
	Unemployment/No work = 5	2944	24.83

**Table 2:** Regression results of the association between Internet use and physical exercise.

	(1)	(2)	(3)	(4)
Variables	Exercise	Frequency of Exercise	Exercise	Frequency of Exercise
	Marginal effects	Coefficients	Marginal effects	Coefficient
Internet use	0.051 ***	0.177 ***		
	(0.011)	(0.037)		
Frequency of Internet use			0.017 ***	0.061 ***
			(0.004)	(0.013)
Age	0.001	0.005 **	0.001	0.004 *
	(0.001)	(0.002)	(0.001)	(0.002)
Gender	−0.018	−0.066	−0.018	−0.066
	(0.012)	(0.039)	(0.012)	(0.039)
Marital status	−0.011	−0.025	−0.011	−0.025
	(0.013)	(0.044)	(0.013)	(0.044)
Hukou	−0.093 ***	−0.334 ***	−0.092 ***	−0.334 ***
	(0.012)	(0.042)	(0.012)	(0.042)
Ethnicity	0.012	0.037	0.012	0.036
	(0.018)	(0.059)	(0.018)	(0.059)
Smoking	−0.016 **	−0.056 **	−0.016 **	−0.056 **
	(0.006)	(0.020)	(0.006)	(0.020)
Drinking	0.012	0.033	0.012	0.034
	(0.009)	(0.031)	(0.009)	(0.031)
Elementary	0.040 ***	0.138 ***	0.040 ***	0.139 ***
	(0.011)	(0.036)	(0.011)	(0.036)
Middle school	0.046 ***	0.167 ***	0.046 ***	0.168 ***
	(0.011)	(0.038)	(0.011)	(0.038)
High school and above	0.079 ***	0.262 ***	0.079 ***	0.262 ***
	(0.014)	(0.047)	(0.014)	(0.047)
Self-assessed health	−0.012 **	−0.036 *	−0.012 **	−0.036 *
	(0.005)	(0.015)	(0.005)	(0.015)
Number of chronic diseases	0.022 ***	0.074 ***	0.022 ***	0.073 ***
	(0.004)	(0.013)	(0.004)	(0.013)
Mental health	−0.003 ***	−0.010 ***	−0.003 ***	−0.010 ***
	(0.001)	(0.002)	(0.001)	(0.002)
Health insurance	0.041	0.128	0.041	0.130
	(0.028)	(0.091)	(0.028)	(0.091)
Pensions	0.032	0.104	0.032	0.104
	(0.019)	(0.063)	(0.019)	(0.063)
Household income	0.005 *	0.017 *	0.005 *	0.018 *
	(0.002)	(0.008)	(0.002)	(0.008)
Employed	−0.022 *	−0.091 *	−0.022 *	−0.090 *
	(0.011)	(0.041)	(0.011)	(0.041)
Retirement	0.100 ***	0.311 ***	0.100 ***	0.311 ***
	(0.019)	(0.058)	(0.019)	(0.058)
Non-farm self-employed	0.006	0.020	0.006	0.021
	(0.013)	(0.045)	(0.013)	(0.045)
Unemployment/no work	0.129 ***	0.402 ***	0.129 ***	0.402 ***
	(0.013)	(0.038)	(0.013)	(0.038)
Provinces	Yes	Yes	Yes	Yes
Pseudo R2	0.100	0.087	0.099	0.088
Observations	11,858	11,858	11,858	11,858

Note: (1) Figures in brackets denote robust standard error; (2) *** *p* < 0.001, ** *p* < 0.01, * *p* < 0.05; (3) Due to space limitation, there is no detailed report on the dummy variables of provinces.

**Table 3:** Regression results of the association between Internet-use purpose and physical exercise.

	(1)	(2)	(3)	(4)	(5)	(6)	(7)	(8)
Variables	Exercise				Frequency of Exercise			
	Marginal effects				Coefficients			
Chat	0.048 ***				0.166 ***			
	(0.012)				(0.041)			
News		0.057 ***				0.206 ***		
		(0.011)				(0.040)		
Video			0.043 ***				0.157 ***	
			(0.012)				(0.042)	
Game				0.023				0.071
				(0.019)				(0.065)
Covariates	Yes	Yes	Yes	Yes	Yes	Yes	Yes	Yes
Provinces	Yes	Yes	Yes	Yes	Yes	Yes	Yes	Yes
Pseudo R2	0.099	0.100	0.099	0.098	0.087	0.088	0.087	0.086
Observations	11,858	11,858	11,858	11,858	11,858	11,858	11,858	11,858

Note: (1) Figures in brackets denote robust standard error; (2) *** *p* < 0.001; (3) Due to space limitation, the regression results of control variables are not reported in detail.

**Table 4:** Regression results of robustness test.

	(1)	(2)	(3)	(4)
Variables	Exercise	Frequency of Exercise	Exercise	Frequency of Exercise
	Marginal effects	Coefficients	Marginal effects	Coefficients
Broadband Internet connection in residence	0.036 ***	0.125 ***		
	(0.009)	(0.029)		
WeChat use			0.050 ***	0.174 ***
			(0.011)	(0.038)
Covariates	Yes	Yes	Yes	Yes
Province	Yes	Yes	Yes	Yes
Pseudo R2	0.099	0.100	0.087	0.088
Observations	11,851	11,851	11,858	11,858

Note: (1) Figures in brackets denote robust standard error; (2) *** *p* < 0.001; (3) Due to space limitation, the regression results of control variables are not reported.

**Table 5:** Intergenerational and urban–rural differences in the association between Internet use and physical exercise.

	(1)	(2)	(3)	(4)	(5)	(6)	(7)	(8)
	Exercise		Frequency of Exercise		Exercise		Frequency of Exercise	
Variables	Middle-Aged Adults	Elderly Adults	Middle-Aged Adults	Elderly Adults	Rural Area	Urban Area	Rural Area	Urban Area
Internet	0.051 ***	0.047 *	0.185 ***	0.155 *	0.042 **	0.068 **	0.172 ***	0.176 **
	(0.012)	(0.020)	(0.046)	(0.066)	(0.013)	(0.022)	(0.050)	(0.057)
Covariates	Yes	Yes	Yes	Yes	Yes	Yes	Yes	Yes
Province	Yes	Yes	Yes	Yes	Yes	Yes	Yes	Yes
	0.105	0.102	0.090	0.091	0.051	0.049	0.046	0.041
Observations	6104	5754	6104	5754	8897	2958	8897	2961

Note: (1) Figures in brackets denote robust standard error; (2) *** *p* < 0.001, ** *p* < 0.01, * *p* < 0.05; (3) Due to space limitation, the regression results of control variables are not reported.

## Data Availability

The data for this study is available from the China Health and Retirement Longitudinal Study (CHARLS) https://charls.charlsdata.com/pages/Data/2018-charls-wave4/zh-cn.html (accessed on 1 September 2022).
